# Localisation of Abundant and Organ-Specific Genes Expressed in *Rosa hybrida* Leaves and Flower Buds by Direct *In Situ* RT-PCR

**DOI:** 10.1100/2012/609597

**Published:** 2012-05-01

**Authors:** Agata Jedrzejuk, Heiko Mibus, Margrethe Serek

**Affiliations:** ^1^Faculty of Natural Sciences, Institute for Ornamental and Woody Plant Science, University of Hannover, Herrenhauser Street 2, 30419 Hannover, Germany; ^2^Department of Ornamental Plants, Faculty of Horticulture and Landscape Architecture, Warsaw University of Life Sciences, Nowoursynowska 166, 02-787 Warsaw, Poland

## Abstract

*In situ* PCR is a technique that allows specific nucleic acid sequences to be detected in individual cells and tissues. *In situ* PCR and IS-RT-PCR are elegant techniques that can increase both sensitivity and throughput, but they are, at best, only semiquantitative; therefore, it is desirable first to ascertain the expression pattern by conventional means to establish the suitable conditions for each probe. In plants, *in situ* RT-PCR is widely used in the expression localisation of specific genes, including MADS-box and other function-specific genes or housekeeping genes in floral buds and other organs. This method is especially useful in small organs or during early developmental stages when the separation of particular parts is impossible. In this paper, we compared three different labelling and immunodetection methods by using *in situ* RT-PCR in *Rosa hybrida* flower buds and leaves. As target genes, we used the abundant **β**-*actin* and *RhFUL* gene, which is expressed only in the leaves and petals/sepals of flower buds. We used digoxygenin-11-dUTP, biotin-11-dUTP, and fluorescein-12-dUTP-labelled nucleotides and antidig-AP/ streptavidin-fluorescein-labelled antibodies. All of the used methods gave strong, specific signal and all of them may be used in localization of gene expression on tissue level in rose organs.

## 1. Introduction

Knowledge regarding the cellular localisation of gene transcripts is essential to assess gene function in an integrated context. There are essentially three different experimental procedures in the field of molecular histology, all of which possess inherent advantages and drawbacks. Promoter-reporter gene fusions may be used to analyse the promoter activity of a target gene [1]. Tissue print RNA hybridisation, based on the transfer of the cytoplasmic contents of fresh tissue sections onto a membrane by hand pressure and subsequent hybridisation with a labelled probe, is an extremely rapid and easy procedure with potential for high-throughput applications [2]. The third and perhaps most widely used method is *in situ* hybridisation (ISH) [3], which may be applied to intact plants (whole-mount *in situ*; [4] or, more classically, to tissue sections [5, 6]). Although procedures based on direct signal visualisation (tissue printing and ISH) have produced a plethora of results, they are essentially limited to target genes with relatively high levels of expression. To overcome this limitation, PCR-based localisation procedures have been established [3, 7], principally with animal tissues and cells, and they are often used in medical applications and to a lesser extent in plants. *In situ* PCR (ISPCR) is a technique that allows specific nucleic acid sequences to be detected in individual cells and tissues [7, 8]. The technique is based on PCR performed on fixed, whole cells or sections; ideally, the PCR product is to be detected at the site of synthesis where it aggregates. Thus far, ISPCR has only been used in animal cells and tissues and predominantly to detect viruses, such as HIV [7–9] or hepatitis C [8, 10].

ISPCR and RT-ISPCR are elegant techniques that can increase both sensitivity and throughput, but they are, at best, merely semi-quantitative [6]; therefore, it is desirable first to ascertain the expression pattern by conventional means to establish the suitable conditions for each probe [11]. *In situ* RT-PCR is a technique that allows the *in situ *visualisation of gene expression at much lower levels than by using *in situ *hybridisation [12, 13]. This technique consists of the reverse-transcription of a targeted RNA within a tissue and the subsequent PCR amplification of the resulting cDNA. Therefore, *in situ *RT-PCR defines a powerful tool for the detection of low-abundance transcripts [6] because the revealing threshold can be as low as one or two copies per cell. In comparison, *in situ *hybridisation detects 10 to 20 copies per cell [14, 15]. The first application of *in situ *RT-PCR for plant tissues was reported by Woo et al. [16] and described the expression of the *HIS 3; 2 *gene (encoding the H1 histone) in single, detached border cells of pea seedlings. The subsequent reports concerning the application of the *in situ *RT-PCR technique to plant material has included several different plants, tissues, and genes [15].

IS-RT-PCR can be further divided into two types, either direct or indirect, based on whether the label is incorporated into the actual PCR product (direct signal detection) [17] or the PCR product is subsequently detected by hybridisation with a labelled probe (indirect detection) [18]. During the direct *in situ *RT-PCR procedure, digoxygenin (biotin or fluorescein) labelled nucleotides [17, 19] or primers [20] are incorporated into the PCR product, leading to a direct signal detection. In contrast, the indirect signal detection for *in situ *RT-PCR occurs when the PCR product is subsequently visualised by hybridisation with a specifically labelled probe [18]. The direct *in situ *RT-PCR technique can be a rapid alternative to the indirect technique because it avoids the subsequent *in situ *hybridisation step [15].

The combination of these two methods, called *in situ* PCR, which was first described by Haase et al. [21], is a highly sensitive technique that is used to localise a single gene copy at the level of individual cells [6, 22].

Since the first successfully optimised *in situ* RT-PCR method was published in 1995 [16], a number of variations on the traditional *in situ *protocols have been reported, including whole-mount ISH (WISH), in-well *in situ *RT-PCR, and the use of vibratome-sectioned tissues [14]. Furthermore, various steps in the tissue preparation and PCR (including sequential pectinase, roteinase, and DNase digestion) have been optimised for *in situ* RT-PCR [11, 17].

In plants, *in situ* hybridisation and *in situ* RT-PCR are widely used in the expression localisation of specific genes, including MADS box and other function-specific genes in floral buds and other organs. This method is especially useful in small organs or during early developmental stages when the separation of particular parts is impossible.

In this report, we present a simplified protocol for *in situ* RT-PCR in the floral buds and leaves of *Rosa hybrida*.

## 2. Material and Methods

### 2.1. Plant Material and Tissue Preparation

The flower buds of *Rosa hybrida* (76/72) are similar to a classic class C-function mutant (flower organs: sepals-petals-petals-sepals) and were selected from an F1 population of the “Lavender Kordana” and “Vanilla Kordana” cultivars (W. Kordes' Rosenschulen Co., Germany) (according to [23]). The plants were propagated from cuttings (four cuttings per pot) under the following greenhouse conditions: temperature at 22°C/18°C (day/night) and a day length extended to 16 h by SON-T lamps (Osram, 400 W, Philips Co.), supplying 600 *μ*mol m^−2^ s^−1^. For restoration of the fertility, the plants were cultivated under conditions of constant humidity at 24°C without assimilation lighting.

Fertile and sterile buds between 2–5 mm in length and mature/young leaves were fixed in PFA fixative (4% paraformaldehyde, 0.4% DMSO, 0.05 M phosphate-buffered saline [PBS, pH 7.0], and DEPC-treated water) or in 4% FAA (4% formaldehyde, 50% ethanol, and 5% glacial acetic acid) for 2 h under a slight vacuum and, subsequently, for 12–24 h at +4°C. Next, the samples were washed twice for 30 min. in PBS, dehydrated in a graded ethanol series (30%, 50%, 70%, 80%, 95%, and 100%) for 1 h in each series at RT (room temperature) under a slight vacuum and twice in histoclear (Histochoice clearing agent, Sigma) for 30 m each time. As a last step before embedding, paraplast pellets (Rotiplast, Roth) were added to the last series of histoclear in a paraffin oven twice a day for 5–7 days at a temperature 56–58°C until the histoclear completely evaporated, and the tissue was embedded in clear paraplast (Rotiplast, Roth).

Semithin sections were prepared in a rotary microtome (Reichert Jung 2040), and the thickness of the preparations ranged between 10–22 *μ*m. All of the preparations were placed on superfrost, RNase-, and DNase-free objective slides (Thermo Scientific MenzelGläser) and dried at 42°C for 2–4 days.

### 2.2. Hydration, Proteinase K, Pectinase, and DNase Treatment

Before the RT step, the slides were dewaxed in histoclear (Histochoice Clearing Agent, Sigma) twice for 10 min and hydrated in a graded ethanol series and PBS. The DNA was digested either with 10 U of DNaseI (Fermentas) in the supplied buffer with 25 mM MgCl_2_ or with 25 mM MnCl_2_ or in a prepared buffer containing 40 mM Tris (pH 7.9), 10 mM NaCl, 6 mM MgCl_2_, and 10 mM CaCl_2_ for 30 min to 8 h at 37°C.

In all cases, the DNaseI was removed by thermal heating at 70°C for 10 m and after rinsing in PBS for 2 m. Optionally, the samples were digested with pectinase (Onozuka) for 10 min at RT. Before the pectinase digestion, the slides were incubated for 2 min in pectinase buffer (0.1 M sodium acetate and 5 mM EDTA, pH 4.5) for 2 min at RT. As a last step, the samples were incubated in proteinase K buffer containing 250 mM Tris-HCl (pH 7.5) and 100 mM Na_2_EDTA and were digested with proteinase K, dissolved in proteinase K buffer (1 mg/mL), for 10–60 min at RT or at 37°C. After the proteinase K digestion, the samples were rinsed in PBS and PBS plus 0.2% glycine and were postfixed in 4% PFA in PBS for 10 min. Before dehydration, the sections were rinsed in 10 mM triethanolamine (Sigma) and 0.25% acetic anhydride (Sigma) for 10 min to reduce the electrostatic binding of the probe during the PCR step; the samples were subsequently dehydrated.

### 2.3. Reverse Transcription and PCR Thermal Cycling

For the reverse transcription step, 50 *μ*L containing 400 U of M-MLV revertase (Promega), the buffer supplied by Promega, 0.5 mM of each dNTP (Roche), 1 *μ*M each of forward and reverse primers and DEPC-treated H_2_O. For the amplification, the following primer pairs were used: *Rh*β*actin* (GenBank: AB239794) forward, 5′-TGCTCCCGCTATGTATGTTG-3′, and reverse, 5′-GGACTTCTGGGCATCTGAAA-3′, and the class A gene *RhFUL* (GenBank:FJ970028) forward, 5′-TCATCCTCCTTTCCCCTTTC-3′, and reverse, 5′-GGACCAGTTTCCCTGTGATT-3′.

The sections were first denatured at 70°C for 5 min and were incubated with the RT reaction mix for 1 h at 42°C. Deactivation of revertase was carried out at 70°C for 10 min.

Immediately after the RT step, the PCR step was carried out in 50 *μ*L containing 0.5 U/*μ*L of DNA polymerase (DNA Cloning Service), Williams buffer, 0.3 mM dNTPs (Roche), 25 mM digoxygenin-11-dUTP (Roche), biotin-11-dUTP (Fermentas) or fluorescein-12-dUTP (Fermentas) and 1 *μ*M of each primer as described above.

The PCR amplification was performed in a thermocycler (Hybaid PCR express) with a flat block under the following conditions: 30 s at 94°C followed by 10, 25, 30, and 40 cycles consisting of 30 s at 94°C for, 1 min at 65°C, 1 min at 72°C, and a final step of 72°C for 10 min. As a negative control, some DNaseI-treated sections were not reverse transcribed in the case of the sections treated with digoxygenin-11-dUTP. The PCR was performed without primers in the case of the biotinylated and fluoresceinated samples. The slides treated with Fluorescein-12-dUTP after the PCR reaction were rinsed in PBS for 2 min, dehydrated, air-dried, and enclosed in mounting medium (Sigma).

### 2.4. Signal Detection

After the PCR step, the samples were denatured in 100% ethanol and stored in PBS overnight at 4°C. The next day, the slides were washed twice in PBS for 30 min at RT, incubated for 1 h in blocking buffer (1% BSA in PBS) and immunoblotted. For the sections treated with Digoxygenin-11-dUTP, immunodetection was carried out with antidigoxygenin-conjugated alkaline phosphatase (Roche) dissolved 1 : 100 in blocking buffer (1% BSA in 1x PBS) for 2 hrs at RT. The samples treated with biotin-11-dUTP were incubated (1 : 20) with FITC-conjugated streptavidin (Sigma, Streptavidin from *Streptomyces avidinii*) primary antibody and, optionally, with antiavidinbiotinylated (Sigma, monoclonal antiavidin—a biotin antibody produced in mice) secondary antibody (1 : 20) for 1 h each at 37°C. After immunodetection with antidigoxygenin, the digoxigenylated samples were washed in blocking buffer and detection buffer (50 mM NaCl, 50 mM Tris-HCl, pH 9.7, and 25 mM MgCl_2_) for 30 min each and were incubated with NBT/BCIP solution (Sigma) diluted 1 : 50 in the detection buffer (50 mM NaCl, 50 mM Tris-HCl, pH 9.7, and 25 mM MgCl_2_) for 30 min to overnight in the dark. After immunodetection, the samples were rinsed in PBS, dehydrated, air-dried, and immersed in mounting medium (Sigma).

The results for the slides treated with fluorescein-12-dUTP and the biotin-11-dUTP-streptavidin-fluorescein system were visualised using an epifluorescence microscopy (Axioscop Zeiss) with a mercury lamp at 50 W (HBO 50/AC and a camera Axio Cam Color 412-312) under an excitation filter of 470–490 nm and under bright-field microscopy (Axioscop Zeiss) and the camera Axio Cam Color 412-312 for the digoxygenin-treated slides.

## 3. Results and Discussion

### 3.1. Tissue Fixation

The first step of the *in situ* transcript localisation involves the preparation of the samples in a way that ensures the optimal preservation of the tissue and cell structure without any deleterious effects on the stability of the RNA [6]. Among the large number of different fixatives, those that are useful for *in situ* techniques may be divided to two groups, specifically, crosslinking and precipitating fixatives. Crosslinking fixatives, such as formalin or (para) formaldehyde and precipitating fixatives, such as simple alcohols and acetone, can give excellent IS-PCR results. Precipitating fixatives are less damaging to nucleic acids but are not as capable of maintaining cellular integrity. For consistent results, the cross-linking fixative should have a neutral pH and be adequately buffered if it is not prepared fresh, the reagents should be of the highest quality, and the length of fixation should not exceed 24 h. An excellent fixative is 4% formaldehyde in a phosphate buffer at pH 7.0–7.4, prepared within 24 h of use. Prolonged fixation offers no advantages and serves only to introduce unwarranted template damage and to extend the permeabilisation steps [24, 25]. For the rose buds, we used two cross-linking fixatives based on 4% FAA and 4% PFA. The PFA was much more efficient with young flower buds (2-3 mm) and young leaves, and a 12-h fixation was sufficient for a good preservation of the tissue, but a 24-h fixation did not damage the tissue. Young organs were too sensitive for FAA, even during a 12-h fixation, which resulted in tissue damage that was noted as cytolysis and cell wall caving. Mature leaves and flower buds (5 mm) fixed with greater integrity in FAA between 12–24 h whereas PFA fixation in these organs resulted in incomplete fixation and lower signal detection. A longer proteinase K digestion (1 h) was necessary. Similar results have been reported by Johansen [8] during the fixation of sugar cane leaves. Even though clear evidence of DNA damage by many fixatives can occur (DNA fragmentation up to 20 kb during glutaraldehyde use and 8–10 kb has been observed during FAA and PFA use) [26], because of the good tissue structure, a much lower incidence of damage occurs when the tissue is stored at 4°C. These fixatives are commonly used for paraffin- or plastic resin-embedded tissue.

### 3.2. DNaseI Digestion and Tissue Permeabilisation

**Figure 1:**
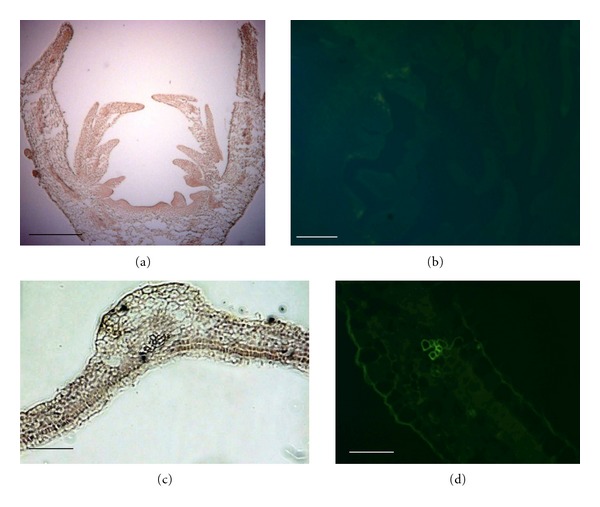
Negative control of *Rosa hybrida *flower buds and leaves (a), (c) Dnase and PCR treated sections. RT step is omitted; (b), (d) PCR-treated sections without primers. Bar = 50 *μ*m.

To avoid the nonspecific binding of primers to DNA, the samples were treated with DNaseI with different buffers that were supplied by the manufacturer (Fermentas) along with Mg^2+^ or Mn^2+^ ions. DNaseI activity is strictly dependent on Ca^2+^ and is activated by Mg^2+^ or Mn^2+^ ions, which cleave the DNA strand in two different ways:

- in the presence of Mg^2+^, DNaseI cleaves each strand of dsDNA independently in a statistically random fashion;
- in the presence of Mn^2+^, the enzyme cleaves both DNA strands at approximately the same site, producing DNA fragments with blunt ends or with overhanging termini of only one or two nucleotides (http://www.fermentas.com/en/home). As a third buffer, we prepared a DNaseI buffer that differed in the Tris concentration (40 mM versus 100 mM from the supplier), the pH (7.9 versus 7.5 from the supplier), and the inclusion of NaCl (in our buffer, 10 mM NaCl was added to stabilise the pH, which is convenient during longer storage). In all cases, DNaseI digested the entire genomic DNA during 30 min at 37°C, which was clearly visible in the sections treated as a negative control with an omitted RT step or with the full RT-PCR procedure but lacking primers in the PCR step (Figures 1(a)–1(d)), and further digestion was unnecessary. In our case, we used 10 U of DNaseI (Fermentas) in 50 *μ*L of the solution, which was sufficient for a 30 min digestion, but other authors recommend longer incubation periods of 3 h to overnight with different concentrations of DNaseI ranging from 4–10 U [6, 15, 17, 19].

For more effective probe penetration during classical *in situ* hybridisation or *in situ* RT-PCR, the samples are permeabilised by proteolytic or, optionally, pectolytic enzymes. In *Rosaceae*, the presence of secondary metabolites, such as polyphenols, tannins, and polysaccharides, may significantly inhibit polymerase activity (according to the work in [27]). To avoid enzyme deactivation by abundant polysaccharides, we used the optional pectinase digestion for 10 min at RT according to Urbanczyk et al. and Przybecki et al. [15, 19] and non-digested slides as a control. Our results did not show any increase in signal, whereas the pectinase was adjusted for comparison of the samples where pectinase digestion was avoided. In this case, we suggest that the polysaccharides present in the *Rosa hybrida* flower buds and leaves did not significantly block the polymerase activity during the *in situ* RT-PCR.

The most crucial and important step in successful *in situ* hybridisation or *in situ* RT-PCR is the proteolytic digestion that makes the crosslinked fixed protein matrix permeable to allow the penetration of the probe or polymerase [28]. The most popular enzymes are proteinase K, pepsin, pepsinogen, and trypsin, and each has its own optimal pH. Until the cellular organisation of DNA or RNA is fully understood, the best enzymes are those with broad substrate specificity. Optimal permeabilisation is largely determined by the type of cell or tissue and the conditions of fixation. Consequently, it is notably difficult to extrapolate these conditions for different samples and protocols, and these conditions should always be determined empirically. In our experiment, we chose proteinase K as the most appropriate proteolytic enzyme for plant tissues [6, 14, 16–18, 29] for 10 to 60 min at RT or 37°C. We noticed that successful proteinase digestion depended on the fixative that was used. PFA-fixed mature leaves and large flower buds (5 mm) required a longer proteinase digestion (up to 60 min) at 37°C. A shorter digestion or a digestion at RT resulted in a weak hybridisation signal. Mature leaves and 5 mm long flower buds fixed in FAA provided the best results with a 30 min digestion at 37°C or 60 min at RT. A 1 h digestion at 37°C resulted in overdigestion and characteristic “bubbles” occurring, especially in the flower buds. The young leaves and small (2-3 mm) flower buds fixed in PFA provided the best digestion results after 30 min at 37°C or RT. The 1-h digestion that was performed under both temperature conditions resulted in tissue damage and the appearance of overdigestion bubbles.

### 3.3. RT-PCR and Signal Detection

**Figure 2:**
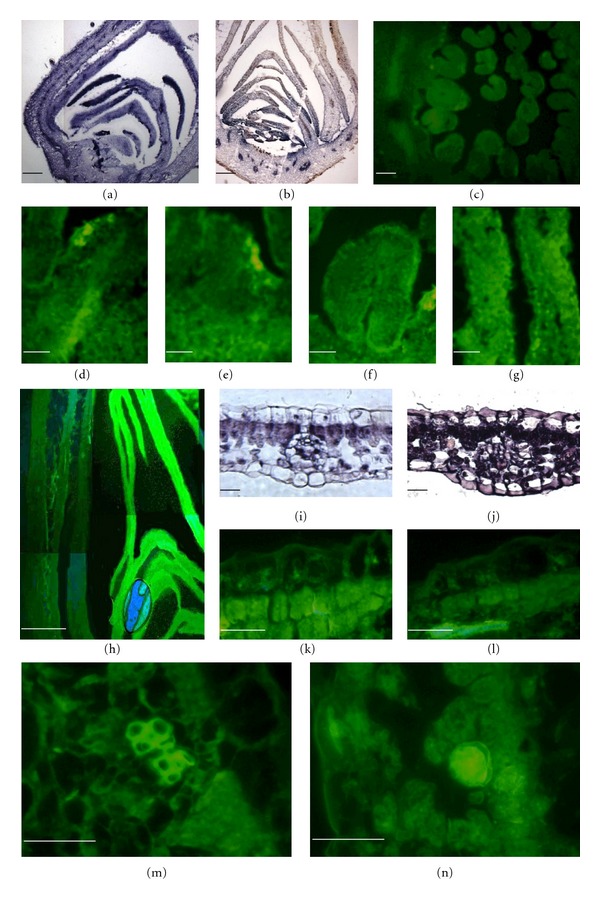
*In situ* RT-PCR on *Rosa hybrida *buds and leaves (a) digoxygenilated sections of flower bud, *β*-actin, (b) digoxygenilated sections of flower bud, Rh FUL gene, (c) biotinylated sections of flower bud, RhFUL gene, (d)–(g) fluoresceinated sections of flower bud, *β*-actin, (h) fluoresceinated sections of flower bud, RhFUL gene, (i) digoxygeninylated sections of leaf, *β*-actin, (j) digoxygeninylated sections, RhFUL gene, (k) biotinylated sections, *β*-actin, (l) biotinylated sections of leaf, RhFUL gene, (m) fluoresceinated sections of leaf, *β*-actin, (n) fluoresceinated sections, of leaf, RhFUL gene. Bar = 50 *μ*m.

For more than 10 years, *in situ* RT-PCR methods have been carried out as a one-step reaction based on the use of rTtH polymerase (Perkin Elmer) [19] or other one-step polymerases. In our experiment, we used M-MLV revertase in the RT step and DCS polymerase in the PCR step. For the RT step, we used specific primers rather than random oligo primers to increase the specificity of the reaction. The samples were first denatured at 70°C for 5 min. Because the last step of the RT reaction was a revertase deactivation at 70°C, which also resulted in cDNA denaturation, the PCR mix was applied to the tissue immediately after the RT reaction, and the PCR reaction was carried out. According to the literature [17, 18, 25, 28, 30–32], there are two basic strategies for labelling the amplified product. One method is to tag the amplicon during the PCR and is generally known as direct IS-PCR or IS-PCR. The direct labelling of the amplicon during the PCR can be accomplished in two ways. The reporter molecule (typically biotin, digoxygenin, or fluorescein) is either attached to a nucleotide (typically dUTP) and added to the PCR or is incorporated during the synthesis of one or both of the primers, usually at the 5′ end. This method of labelling is the easiest way, but it may result in a false-positive signal [33–35]. Strategies for inhibiting this nonspecific incorporation, including hot start, 3′ to 5′ exonuclease-deficient DNA polymerases, and capping, have proven unsuccessful [14, 34]. Although labelled oligonucleotides may provide problems with false-positive signals as unspecific labelling or unspecific background as cytoplasm staining, most of the *in situ* PCR procedures recommend this type of labelling. The unspecific labelling may be reduced by carefully optimising the annealing temperature of the PCR and confirming the specificity of the signal by performing a parallel indirect IS-PCR. In our experiment, we performed three different labelling and signal detection methods during the direct *in situ* RT-PCR. For the labelled nucleotides, we used digoxygenin-11-dUTP, which was immunolabeled with alkaline phosphatase-bound antidigoxygenin and NBT/BCIP solution, biotin-11-dUTP, which was immunolabeled with streptavidin-fluorescein and, optionally, antiavidin biotinylated to strengthen the signal. As the most direct *in situ* RT-PCR method, we used fluorescein-12-dUTP as one of the labeled nucleotides and the same, omitted long immunolocalisation procedure. As a target gene, we chose the abundant **β*-actin* that is highly expressed in all organs, including flower buds and leaves, and an MADS box-specific class A gene, which is highly expressed in leaves and only in the sepals/petals of flower buds, to confirm the specificity of the signal detection. We also used flower buds and leaves in different developmental stages to determine the intensity of the product amplification during the immunolocalisation. To achieve successful results, the most important variables during the PCR reaction are the concentrations of Mg^2+^, a thermostable DNA polymerase, the primers, the annealing temperature, and the cycle number. During the PCR step, we strictly maintained the principle of not exceeding 40 cycles. In theory, 10 PCR cycles should generate enough signal for detection if the amplification is close to exponential [36, 37], but, in our case, even during the amplification of such an abundant gene as *β-actin* in young flower buds and leaves, 10 cycles were insufficient; we found that 25 cycles were sufficient for all of the labelling methods (Figure 2(a)–2(n)). When we used an organ-specific gene (*RhFUL* gene), a clear signal was evident after 30 cycles in the labelling with digoxygenin/fluorescein, but nonspecific binding appeared in the biotin-labelled samples even with only 10 PCR cycles. Consequently, the recommendations for the thermal and biochemical parameters for performing PCR on slides can only be described in general, and a brief inspection of the literature reveals little uniformity in the published protocols [14, 35, 38].

It has been reported that streptavidin can bind to biotin-containing proteins in tissue, resulting in nonspecific signals [16, 39], which is a situation that is not observed when digoxygenin or fluorescein are used as labels. Another point may be that during the thermal cycling, some of the proteins may be denatured, which may also cause unspecific binding of the antibody during immunolocalisation. During signal detection, we tested antidigoxygenin AP Fab fragments (Roche antibody) for digoxygenin, streptavidin-fluorescein, and, optionally, the antiavidin-biotin system for biotin.

The results of our experiment clearly showed that the most specific binding was achieved when we used digoxygenin and fluorescein as labels. The signal was similarly strong in the tissues where *β*-actin was localised and, specifically, in the petals whereas the target (*RhFUL*) gene was localised in the flower buds (Figures 2(a), 2(b), 2(d)–2(j), 2(m) and 2(n)). The results of our investigations showed unspecific binding of streptavidin-fluorescein to all of the organs in the flower bud when the *RhFUL* gene was used (Figure 2(c)). This background was probably caused by the unspecific binding of streptavidin to the endogenous biotin present in the *Rosa hybrida* buds, although there is insufficient data about the natural biotin content in rose organs, especially in the leaves and flower buds.

Special attention should be paid to the Primed *in situ* DNA labelling (PRINS) method, which was first described by Koch [39] as the most indirect method of *in situ* RT-PCR, in which one of the oligonucleotides is fluorochrome-labelled, thereby rendering further immune detection unnecessary.

PRINS is widely used during the localisation of repetitive and telomeric sequences in plant chromosomes [40, 41], but it is not sensitive enough for the localisation of particular genes. Our results showed that direct *in situ* PCR with use of fluorescein-12-dUTP as a labelled nucleotide gives comparable signal strength and specificity as PCR labelled with digoxygenin and may be used to detect abundant and site-specific gene expression. Roses are one of the most economically important groups of ornamental plants, and a number of varieties have been selected based on flower traits, such as petal form, colour, and number [42, 43]. The highest level of interest by researchers and breeders is regarding flower colour and the number of petals, which is connected to MADS-box genes expression, particularly flower organs. For several years, *in situ* RT-PCR has commonly been used in the localisation of the expression of different genes in different tissues in herbaceous and woody plants. According to many literature resources, successful results have been achieved by *in situ* RT-PCR, even in the vascular tissue of such woody plants as *Populus tremula* [43]. According to the literature [14, 16, 18, 25, 28, 29, 35], the* in situ* RT-PCR method is mostly used for the localisation of the expression of abundant genes, such as those responsible for expansin activation or virus-associated genes [43–45]. In much of the available literature, specific-function genes, such as MADS-box genes, in herbaceous and woody plants are mostly localised by traditional *in situ* hybridisation (e.g., an *AGAMOUS* homolog in black spruce [46], an MADS-box family gene in Monterey pine [47], an MADS-box family gene in eucalyptus [48], a *DEFICIENS* homologue [49], and an MADS-box family gene in apple [50, 51]. In this report, we presented a convenient protocol for the localisation of transcript expression in the different organs of *Rosa hybrida*. The protocol is more appealing because of a high sensitivity for the *in situ* RT-PCR reaction and its speed. We demonstrated that a two-step reaction can be completed in two days, and a one-step reaction with fluorescein-12-dUTP used as a label can be completed in one day. Another convenience is the avoidance of the probe preparation. Our results showed that a normal RT-PCR reaction performed directly on tissue showed a high specific expression of the chosen genes, namely, the abundant and widely expressed *β*-actin and sepal/petal-specific *RhFUL* gene.

## 4. Conclusions

In this report, we compared three different labelling and immunodetection methods by using *in situ* RT-PCR in *Rosa hybrida* flower buds and leaves. As target genes, we used the abundant **β*-actin* and *RhFUL* gene, which is expressed only in the leaves and petals/sepals of flower buds. We used digoxygenin-11-dUTP, biotin-11-dUTP, and fluorescein-12-dUTP-labelled nucleotides and antidigoxygenin-alkaline phosphatase/streptavidin-fluorescein labeled antibodies.

We conclude that 25 PCR cycles are sufficient for clear evidence of abundant gene expression and 30 cycles are sufficient for site-specific genes.

The fastest method of transcript localisation in leaves and flower buds is direct PCR with fluorescein used as one of the labelled nucleotides. The highest signal sensitivity was achieved using digoxygenin-11-dUTP or fluorescein-12-dUTP as the labelled nucleotides. The biotin-streptavidin labelling system failed because of the unspecific background associated with the localisation of *RhFUL* gene expression in the flower buds. The chosen fixatives (4% PFA and 4% FAA) confirmed the general thesis that PFA preservatives work better in young tissue. We also optimised the digestion conditions and enzyme concentrations for DNaseI and proteinase K. We proved that the optional digestion of pectins is not required to achieve clear and strong signals in rose buds and leaves during *in situ* RT-PCR.
